# The Prevalence of Risk Factors Among Children Diagnosed With Attention-Deficit/Hyperactivity Disorder, Aged 4–17 Years: A Cross-Sectional Study

**DOI:** 10.7759/cureus.49161

**Published:** 2023-11-21

**Authors:** Abeer F Zakariyah

**Affiliations:** 1 Department of Medical Genetics, Faculty of Medicine, University of Jeddah, Jeddah, SAU

**Keywords:** saudi arabia, cross-sectional study, children, risk factors, adhd, prevalence

## Abstract

Introduction: Attention-deficit/hyperactivity disorder (ADHD) is a neurodevelopmental disorder commonly observed in children. Although the etiology of ADHD is still unclear, many risk factors have been shown to increase the prevalence of ADHD, such as genetics, environmental factors, socioeconomic status, maternal smoking, and low birth weight.

Aim: The current cross-sectional study aimed to assess the prevalence of several risk factors associated with ADHD-diagnosed children aged 4-17 years using parent-reported data.

Methods: An observational cross-section study was conducted between December 2022 and February 2023 using a self-constructed questionnaire that we sent to parents whose children were diagnosed with ADHD. The questionnaire included socioeconomic information (income, gender, parent education, number of children), child information (age, ADHD type, academic achievement), pregnancy and neonatal period (smoking status, gestation age, mode of delivery, child weight), and if there is a history of ADHD in the family.

Results: A total of 306 parents responded to the questionnaire. The majority of our study population was males (77.8%). The combined type, which includes symptoms of both hyperactivity and inattention, represented 70% of the population in our study, which is higher than the inattention type (23%) and the hyperactivity type (6%). The prevalence of several risk factors known to be associated with ADHD, such as family income, parental education, complications during pregnancy, and the low birth weight of the child, were also reported.

Conclusion: To the best of our knowledge, this is the first study that examines the frequency of risk factors among children diagnosed with ADHD in Saudi Arabia. The study revealed that males are more likely to be diagnosed with ADHD than females. We also found that the combined type is the most diagnosed ADHD among children. Furthermore, there are no significant differences in the prevalence of the risk factors during the gestational or neonatal period among ADHD-diagnosed children. Therefore, a large-scale prospective study is needed to aid in evaluating the frequency and significance of various risk factors among diagnosed children

## Introduction

Attention-deficit/hyperactivity disorder (ADHD) is a neurodevelopmental condition that manifests during childhood and may persist throughout adulthood [1]. It is characterized by attention deficits, hyperactivity, and impulsive behavior. It is known that ADHD has the potential to impact an individual's academic performance, social interactions, and occupational abilities [2].

The Diagnostic and Statistical Manual of Mental Disorders, Fifth Edition (DSM-5) defines three classifications of ADHD [3]. The first type is known as hyperactive/impulsive, and the second is the inattentive type, distinguished by inattention symptoms without hyperactivity or impulsivity. The third type is the combined type, which represents the most prevalent form of ADHD and is characterized by symptoms of both inattention and hyperactivity/impulsivity [3]. Although ADHD can be diagnosed in adults, it is most commonly identified in children when they start school. This early identification allows for interventions to minimize social and educational disabilities related to the disorder [4]. The American Academy of Pediatrics (AAP) advises diagnosing ADHD in children aged 4-18 years [5].

Multiple published systematic reviews and meta-analyses have offered valuable information regarding the prevalence of ADHD in children and adolescents. The global prevalence of persistent adult ADHD in 2020 was 2.58%, affecting about 139.84 million individuals worldwide [6]. Also, a systematic review showed that Europe's prevalence of ADHD in children and adolescents ranges from 5.9% to 7.1%, depending on the diagnostic criteria used [7]. In addition, a systematic review conducted in Arab countries revealed a wide range in the prevalence of ADHD, with rates ranging from 1.3% to 16% [8]. The most recent systematic review in Saudi Arabia found that the prevalence of ADHD is 12.4%, like in other countries in the Middle East and North Africa [9].

Many studies have been undertaken to comprehend the etiology of ADHD, although the precise cause of this condition remains unknown. However, prior research has linked various factors, including environmental and genetic factors to ADHD [10,11]. Many studies have demonstrated that variables like gender, paternal education, and socioeconomic status are related to ADHD [12,13]. Furthermore, there has been significant interest in investigating the impact of specific gestation and perinatal-related risk factors on the development of ADHD. For example, maternal stress, anxiety, depression, preeclampsia, smoking during pregnancy, and low birth weight are significantly associated with ADHD, as indicated by several studies [14-20].

Examining the prevalence of existing contributing factors in the diagnosed cases would be advantageous. This study aimed to determine the diverse sociodemographic variables linked to the diagnosis of ADHD in children and adolescents aged 4-17 years. Furthermore, it assesses the frequency of factors such as maternal complications during pregnancy, child weight, and family history. To the best of our knowledge, this study is the first to specifically examine the prevalence of risk factors among children diagnosed with ADHD. Hence, the results of this investigation will aid in evaluating the frequency of various factors among children with ADHD who reside in Saudi Arabia. These characteristics can serve as predictors of ADHD, aiding in the early detection and intervention of the condition.

## Materials and methods

Thiswas an observational cross-sectional study conducted between December 1, 2022, and February 28, 2023. The study was approved by the Bioethics Committee and Institutional Review Board at Jeddah University, Jeddah, Saudi Arabia (approval number: UJ-REC-080). Throughout the study, confidentiality was upheld.

Questionnaire

A self-constructed validated questionnaire for this study contains demographic information and closed-ended questionnaires. To validate the questionnaire, it was first administered to a cohort of mothers to assess its comprehensibility and clarity. Then, the responses from the group were examined using the Cronbach test to ascertain the reliability and validity of the questions. The Cronbach's alpha test confirmed that the questions had a satisfactory reliability coefficient of 0.6. The questionnaire comprised four parts. The first set of variables consisted of sociodemographic factors, including age, gender, region of residence, family income, number of children, and the parent's educational level, ranging from primary school to PhD level. The second part comprised data related to the child, including their age, type of ADHD, academic performance, and their order among their siblings. The third part included inquiries regarding whether they have a family history of ADHD (yes or no). The fourth section comprised questions regarding the pregnancy and perinatal period, including the mother's health condition during pregnancy, such as diabetes, hypertension, and preeclampsia; smoking habits; the method of delivery (natural birth or C-section); as well as the gestational age and birth weight of the child. See the Appendices for the questionnaire used in the current study.

Participants and data collection

All parents of children aged 4-17 years who were diagnosed with ADHD and registered with Eshraq Society (https://adhd.org.sa/en/) were included in the study. Eshraq is a society that provides aid and encouragement to individuals who have received a diagnosis of ADHD. Approximately 900 children aged 4-17 years were registered with the Eshraq Society and diagnosed with ADHD. The sample size was calculated using the OpenEpi software, based on the most recent systematic review and meta-analysis on the prevalence of ADHD in Saudi Arabia at 12% [9]. The sample size was concluded to be 163 at a 95% confidence limit. Data was collected using an online questionnaire in Google Forms (Google LLC, Mountain View, California, United States) and distributed through the Eshraq society's Twitter (X) account (X Corp., San Francisco, California, United States), email, and SMS. The participants' filling out the questionnaire signifies their informed consent and willingness to engage in the study. The participants were required to respond to various sections of the questionnaire. Out of the 900 parents who were given the questionnaire, 389 parents willingly decided to take part and successfully filled out the questionnaires, resulting in a response rate of 43%. Individuals excluded from the study were those who did not have a medical diagnosis of ADHD (17.7%, n = 69) or were children under the age of 4 (3.6%, n = 14). Therefore, our current study involved a total of 306 participants.

Data analysis

After extracting the data from Google Forms, the information was entered and coded in an Excel sheet (Microsoft Corporation, Redmond, Washington, United States). Subsequently, the data was analyzed using Microsoft Excel and IBM SPSS Statistics for Windows, Version 23.0 (Released 2015; IBM Corp., Armonk, New York, United States). Descriptive statistics were computed for all variables. The prevalence has been quantified as a percentage. The categorical variables are displayed as percentages of frequency. The chi-square test was used to compare categorical variables, and the significance level was established at a p-value of less than 0.05.

## Results

Sociodemographic characteristics of the families of children diagnosed with ADHD

A total of 306 parents whose children (aged 4-17 years) were diagnosed with ADHD responded to the questionnaire. As indicated in Table 1, the majority of the participants were from the central region of Saudi Arabia (56.2%, n = 172). Most participants (43%, n = 132) had a monthly income of more than 10,000 Saudi Riyals (SAR). Regarding the number of children, most families reported having three kids (28.4%, n = 87). Most parents who participated in the study and had a child diagnosed with ADHD have completed a bachelor’s level of education. More than half of the mothers (53.3%, n = 163) had bachelor’s degrees. Most fathers (45.4%, n = 136) also had a bachelor’s degree.

**Table 1:** Sociodemographic and family-related characteristics of children covered in the study (n=306).

Variables		Frequency, n=306	Percentage (%)
Region	Central Region	172	56.2
	Western Region	92	30.1
	Eastern Region	19	6.2
	Northern Region	11	3.6
	Southern Region	12	3.9
Family income	More than 10,000 SAR	132	43.1
	From 5000-10,000 SAR	105	34.3
	Less than 5000 SAR	69	22.5
Number of siblings	One	31	10.1
	Two	66	21.6
	Three	87	28.4
	Four	66	21.6
	Five or more	56	18.3
Mother's educational level	PhD	15	4.9
	Master	30	9.8
	Bachelor	163	53.3
	Diploma	8	2.6
	Secondary school	58	19.0
	Elementary school	15	4.9
	Primary school	17	5.6
Father's educational level	PhD	15	4.9
	Master	38	12.4
	Bachelor	139	45.4
	Diploma	20	6.5
	Secondary school	66	21.6
	Elementary school	17	5.6
	Primary school	11	3.6

Characteristics of children diagnosed with ADHD

Most children in the study group (47.7%, n = 146) were in the age group of 7-10 years, followed by 11-12 years (24.8%, n = 76), and 4-6 years (20.9%, n = 64). We also found that most diagnosed children were males (77.8%, n = 238). In addition, almost three-fourths of the parents (70%, n = 215) reported their children being diagnosed with the combined type. Regarding the academic achievements of the children, most parents reported that their children had a good level at school (57.5%, n = 176), followed by acceptable (24.2%, n = 74) and excellent (18.3%, n = 56). Also, most children diagnosed with ADHD were the first child born in the family (43%, n = 132), followed by the second (20%, n = 62), and the third (14.3%, n = 44). Table 2 shows the characteristics of the children in the current study.

**Table 2:** The frequency of age, gender, ADHD type, and academic achievement of the children covered in the study (n=306). ADHD: attention-deficit/hyperactivity disorder

Children's information (Variables)		Frequency, n=306	Percentage (%)
Child Age	4-6 years	64	20.9
	7-10 years	146	47.7
	11-12 years	76	24.8
	13-17 years	20	6.5
Gender	Female	68	22.2
	Male	238	77.8
ADHD type	Inattentive type	72	23.5
	Hyperactive type	19	6.2
	Combined types	215	70.3
Academic achievements	Excellent	56	18.3
	Good	176	57.5
	Acceptable	74	24.2
Child order	First	132	43.1
	Second	62	20
	Third	44	14.3
	Fourth	40	13
	Fifth and last	28	9.1

The prevalence of risk factors among the children diagnosed with ADHD

Since all participants in the study are diagnosed with ADHD, we wanted to determine the prevalence and significance of several factors such as gender, parent's education, family income, family history, and pregnancy and neonatal periods among the participants in our study. We found that the prevalence of diagnosed males (77.8%, n = 238), p = 0.003, was significantly higher than that of females. We also found that parents' education level among children diagnosed with ADHD was higher at a bachelor’s degree than at other levels of education, but there were no significant differences. A total of 89 (30%) diagnosed children reported having a family history of ADHD. Regarding the family income, the parents who reported having an income of more than 10,000 SAR were the highest (43%, n = 132) compared to other incomes, as shown in Table 3.

We also asked the parents about the pregnancy period, including any complications and whether the mother smoked or was exposed to smoking during pregnancy, as shown in Table 3. Most mothers of the diagnosed children (81.4%, n = 249) reported that they did not have any complications during pregnancy. However, some of the mothers reported they had hypertension (4.6%, n = 14), diabetes (12.4%, n = 38), and preeclampsia during pregnancy (1.6%, n = 5). Also, few of the mothers (5.2%, n = 16) reported smoking during pregnancy, and 39.5% (n = 121) reported exposure to smoking.

We also asked the parents about the gestational age, type of delivery, and the child's weight, as shown in Table 3. While some parents do not remember the gestational age, most parents (37.9%, n = 116) reported that their children were born at 37-39 weeks, while few reported (4.6%, n = 14) that their children were born at less than 36 weeks. Regarding the delivery type, more than half (67.3%, n = 206) were born naturally, while 32.7% (n = 100) were C-sections. Lastly, most children diagnosed with ADHD in the current study (43.8%, n = 134) weighed 2.5-3.5 kg, followed by 23.5% (n = 72) born at 2-2.5 kg.

**Table 3:** Prevalence of child gender, parent education, family income, family history, pregnancy, and neonatal period details among the participants

Variable		n = 306, percentage (%)	P -value
Family information	Child gender		
	Female	68 (22.2%)	0.003*
	Male	238 (77.8%)	
	Father education		
	Primary school	11 (3.6%)	0.06
	Intermediate school	17 (5.6%)	
	High school	66 (21.6%)	
	Diploma	20 (6.5%)	
	Bachelor	139 (45.4%)	
	Master	38 (12.4%)	
	PhD	15 (4.9%)	
	Mother education		
	Primary school	17 (5.6%)	0.95
	Intermediate school	15 (4.9%)	
	High school	58 (19.0%)	
	Diploma	8 (2.6%)	
	Bachelor	163 (53.3%)	
	Master	30 (9.8%)	
	PhD	15 (4.9%)	
	Family history of ADHD		
	Yes	89 (29.1%)	0.34
	No	217 (70.9%)	
	Family income		
	< 5000 SAR	69 (22.5%)	0.14
	5000-10,000 SAR	105 (34.3%)	
	>10,000 SAR	132 (43.1%)	
Pregnancy period	Did the mother suffer from any complication		
	Hypertension	14 (4.6%)	0.41
	Diabetes	38 (12.4%)	
	Preeclampsia	5 (1.6%)	
	Nothing	249 (81.4%)	
	Did the mother smoke		
	Yes	16 (5.2%)	0.26
	No	290 (94.8%)	
	Was the mother exposed to smoke		
	Yes	121 (39.5%)	0.58
	No	185 (60.5%)	
Neonatal period	Gestational age		
	< 36 Weeks	14 (4.6%)	0.21
	37-39 Weeks	116 (37.9%)	
	40-42 Weeks	93 (30.4%)	
	Don't remember	83 (27.1%)	
	Delivery		
	C-Section	100 (32.7%)	0.44
	Natural	206 (67.3%)	
	Child weight		
	> 3.5 kg	41 (13.4%)	0.28
	2.5kg-3.5kg	134 (43.8%)	
	2 kg 2.5kg	72 (23.5%)	
	< 2 kg	23 (7.5%)	
	Don't remember	36 (11.8%)	

## Discussion

The precise etiology of ADHD remains poorly understood; however, existing knowledge suggests that it results from a combination of genetic risk and environmental factors. In the present investigation, we conducted a cross-sectional study distributed to parents whose children are diagnosed with ADHD aged 4-17 years. The current study focused on determining the prevalence of risk factors that are known to be associated with ADHD. No significant differences were found in the sociodemographic characteristics or the risk factors during pregnancy or the neonatal periods among ADHD-diagnosed children. Nevertheless, it has been seen that the male-to-female ratio (3:1) is remarkably elevated among the participants in the study.

Several studies have addressed gender and sex differences in ADHD. Our findings indicate a significant gender difference in ADHD prevalence, with a higher incidence observed in males than females. This aligns with previous research that consistently reported a higher frequency of ADHD symptoms in boys than in girls [21]. Hence, there is a notable disparity between males and females regarding ADHD. This discrepancy may arise from the fact that females are frequently subject to underdiagnosis or misdiagnosis due to differences in symptoms [22]. Studies also indicated that the lack or excess of an X chromosome can impact ADHD cognition and behavior. A study showed that X chromosome variation may explain ADHD prevalence discrepancies between males and females. Therefore, X-linked gene dosage or genetic variations may explain why ADHD is more common in males than females [23]. We also found that ADHD was more common at younger ages, as shown in a previous study where ADHD was more prevalent when children started school [21,24].

Many studies have demonstrated an association between those of lower socioeconomic status and a greater prevalence of ADHD [25]. Nevertheless, it is essential to highlight that in our study, a higher percentage of children diagnosed with ADHD originated from households with a family income beyond 10,000 SAR. Therefore, our study showed no apparent correlation between low family income and ADHD, which is also aligned with a systematic review that found no association [26]. Also, several studies showed that a low level of parental education is associated with a higher risk of ADHD in offspring [27,28]. The current study found that most parents with diagnosed children had a bachelor's degree, and there was no association between a low level of education and an increased chance of ADHD in their offspring. This finding is similar to another study conducted in the United Arab Emirates, where they showed no association between the low level of parent’s education and the increased risk of ADHD in their children [29]. The differences in findings among the studies may be attributed to differences in healthcare and education systems among countries. For instance, increased accessibility to resources and enhanced ability to access medical systems, including seeking additional second opinions and owning prior knowledge about ADHD.

Studies suggest that the parental origin of genetic risk factors may contribute to the etiology of ADHD [30]. Nevertheless, the precise mechanism of inheritance remains unidentified. The current study found that one-third of the parents reported having a family history of ADHD. The risk of genetic factors in the family with other risk factors could increase the chances of ADHD in offspring. Further studies are needed to understand the association between family history and other risk factors.

Complications such as preeclampsia and hypertension, as well as maternal smoking during pregnancy and low birth weight, are risk factors that have been investigated and reported to increase the risk of ADHD in children [14,16,17]. Most mothers in our study reported that they did not have complications during pregnancy. Moreover, we also noted that while a very low percentage of mothers reported smoking during pregnancy, 40% were exposed to it. Also, we found that most diagnosed children were born within the normal weight range of 2.5-3.5 kg. Therefore, further investigation is needed with a larger sample size to address the association between ADHD and other factors such as family history, maternal smoking, and low birth weight.

It is important to note several limitations of the current study. Initially, the small quantity of samples limited the ability to obtain significant results. Therefore, a larger cohort study is required to examine the relationship between risk factors occurring during pregnancy and ADHD. Furthermore, the study's reliance on self-reporting may lead to recall bias. It is essential to investigate the impact of several risk factors in relation to ADHD. Another limitation is that the study was observational and did not include a control group. Lastly, it has been demonstrated that psychological problems such as stress and depression are linked to an increased likelihood of ADHD in offspring. However, we did not investigate this association in the present study. Further studies with a larger sample of diagnosed children are needed to investigate the association between several risk factors and ADHD.

## Conclusions

This cross-sectional study aimed to assess the frequency of risk factors associated with ADHD among children aged 4-17 diagnosed with the disorder. The current study demonstrated a higher prevalence of ADHD in males than females. It is also more prevalent among children in lower grades. Furthermore, the combined type of ADHD was the most common. In addition, the study showed no notable differences in the sociodemographic characteristics or other risk factors during the pregnancy or neonatal period among children diagnosed with ADHD. This work contributes to the current understanding that the exact cause of ADHD is still not well understood, although it arises from a mix of genetic susceptibility and environmental influences. This study also emphasizes the necessity for additional research on a larger scale to determine the risk factors associated with ADHD and to develop appropriate preventative and therapeutic strategies.

## Appendices

Questionnaire

Consent to Participate in the Questionnaire

1.     Do you agree to participate in the questionnaire?

-       Yes

-       No         (exit from the questionnaire)

2.     A specialized health professional diagnosed your child with ADHD.

-       Yes

-       No     (exit from the questionnaire)

Section 1:

3.     Age of the mother: _____

4.     Age of the father:______

5.     Nationality

-       Saudi

-       Non-Saudi

6.     Region of residence

-       Central

-       Northern

-       Southern

-       Western

-       Eastern

7.     The last mother’s educational level

-       Primary school

-       Intermediate school

-       Secondary school

-       Diploma

-       Bachelor’s degree

-       Master’s degree

-       PhD degree

8.     The last father’s educational level

-       Primary school

-       Intermediate school

-       Secondary school

-       Diploma

-       Bachelor’s degree

-       Master’s degree

-       PhD degree

9.     Family income

-       Less than 5000 SAR

-       From 5000 to 10,000 SAR

-       More than 10,000 SAR

10.  Number of children in the family.

-       1

-       2

-       3

-       4

-        Five and more

Section 2:

11.  What is the gender of your child diagnosed with ADHD?

-       Male

-       Female

12.  What is the age of your child diagnosed with ADHD?

-       Under four years

-       4-6 years

-       7-10 years

-       11-12 years

-       13-17 years

13.  What is the order of your child diagnosed with ADHD?

-       First

-       Second

-       Third

-       Fourth

-       Fifth and last

14.  The type of ADHD your child is diagnosed with is:

-       Hyperactive type

-       Inattentive type

-       Combined type

15.  Your child’s level in school

-       Excellent

-       Good

-       Acceptable

Section 3:

16.  Does your child have a family history of ADHD

-       Yes

-       No

17.  If the answer is yes, what is the relationship to the child?________

Section 4:

18.  The type of delivery

-       Natural

-       C-section

19.  The gestation period.

-       Less than 36 weeks

-       37-39 weeks

-       40-42 weeks

-       I do not remember.

20.  The child’s weight upon delivery

-       Less than 2 kg

-       2 kg-2.5 kg

-       2.5kg-3.5 kg

-       More than 3 kg

-       I don’t remember.

21.  Did the mother suffer from the following during pregnancy?

-       Preeclampsia

-       Diabetes

-       Hypertension

-       Did not suffer from anything.

22.  Did the mother smoke during pregnancy?

-       Yes

-       No

23.  Was the mother exposed to smoking during pregnancy?

-       Yes

-       No
